# Morphological Changes and Cellular Uptake of Functionalized Graphene Oxide Loaded with Protocatechuic Acid and Folic Acid in Hepatocellular Carcinoma Cancer Cell

**DOI:** 10.3390/ijms21165874

**Published:** 2020-08-16

**Authors:** Kalaivani Buskaran, Mohd Zobir Hussein, Mohamad Aris Mohd Moklas, Sharida Fakurazi

**Affiliations:** 1 Laboratory for Vaccine and Immunotherapeutic, Institute of Biosciences, Universiti Putra Malaysia, Serdang, Selangor 43400, Malaysia; vaneey_88@yahoo.com; 2 Materials Synthesis and Characterization Laboratory, Institute of Advanced Technology, Universiti Putra Malaysia, Serdang, Selangor 43400, Malaysia; mzobir@upm.edu.my; 3 Department of Human Anatomy, Faculty of Medicine and Health Sciences, Universiti Putra Malaysia, Serdang, Selangor 43400, Malaysia; aris@upm.edu.my

**Keywords:** graphene oxide, protocatechuic acid, cellular uptake, drug delivery system

## Abstract

The development of nanocomposites has swiftly changed the horizon of drug delivery systems in defining a new platform. Major understanding of the interaction of nanocomposites with cells and how the interaction influences intracellular uptake is an important aspect to study in order to ensure successful utilisation of the nanocomposites. Studies have suggested that the nanocomposites’ ability to permeate into biological cells is attributable to their well-defined physicochemical properties with nanoscale size, which is relevant to the nanoscale components of biology and cellular organelles. The functionalized graphene oxide coated with polyethylene glycol, loaded with protocatechuic acid and folic acid (GOP-PCA-FA) nanocomposite intracellular uptake was analysed using transmission electron microscope. The accumulation of fluorescent-labelled nanocomposites in the HepG2 cell was also analysed using a fluorescent microscope. In vitro cellular uptake showed that there was uptake of the drug from 24 h into the cells and the release study using fluorescently tagged nanocomposite demonstrated that release and accumulation were observed at 24 h and 48 h. Moreover, the migration ability of tumor cells is a key step in tumor progression which was observed 48 h after treatment. The GOP serves as a potential nanocarrier system which is capable of improving the therapeutic efficacy of drugs and biomolecules in medical as well as pharmaceutical applications through the enhanced intracellular release and accumulation of the encapsulated drugs. Nonetheless, it is essential to analyse the translocation of our newly developed GOP-PCA-FA, and its efficiency for drug delivery, effective cellular uptake, and abundant intracellular accumulation would be compromised by possible untoward side effects.

## 1. Introduction

Engineered nanocomposites as a nanomedicine are a growing innovative field which attracts the attention of biomedical scientists worldwide. However, there still remains some uncertainty particularly about the mode of action of the manufactured nanocomposites. Countless studies have suggested that the nanoscale dimension of the nanocomposites favour access into the biological environment, especially the human body [1,2]. The exclusive physicochemical properties of the nanocomposite, making it so desirable in drug delivery. However, it might also contribute to possible cytotoxicity. Therefore, biological evaluation of nanocomposites in the cells and tissues is essential especially when the nanocomposite has intended medical applications. In the drug delivery system, the first criteria to take into account during nanocomposite and cell interaction is the route of entry of the nanocomposite into the cell. Basically, nanocomposites enter the cells by different mechanisms, such as phagocytosis, macropinocytosis, endocytosis, or directly by so-called adhesive interactions [3].

The drug delivery system and cellular response are correlated to the accumulation and internalization of the nanocomposites into the cells. Meanwhile, the accumulation, quantity, and degradation of the nanocomposites within intracellular compartments and its impact on cell viability can be observed using the transmission electron microscope (TEM). Observation using TEM known as the gold standard for capturing the details of ultrastructural changes with the best illustrations [4]. Fluorescein isothiocyanate (FITC) conjugated nanocomposite is capable of identifying the accumulation of nanocomposites in the cells. The migration of HepG2 cells was assessed using cell exclusion zone assay. The efficient delivery of nanocomposites into cell interiors is crucial to ensure the success of a treatment.

In the previous reported study by Saifullah et al. 2018, an anticancer nanocomposite delivery system for protocatechuic acid using graphene oxide–polyethylene glycol as the nanocarrier was designed. The nanocomposite was tagged with folic acid (GOP–PCA–FA) intended for active targeting of cancer cells. The cytotoxicity activity of the GOP-PCA-FA (18.89 μg/mL) nanocomposite was better than that of GOP-PCA (29.98 μg/mL) and pristine PCA (38 μg/mL) against HepG2 (human hepatocellular carcinoma cell). The nanocomposite exhibited nontoxicity or less cytotoxicity in healthy fibroblast 3T3 cells. Coating the nanocomposite with folic acid showed a better cytotoxicity activity compared to the uncoated system [5]. Subsequently, it is essential to investigate the cellular uptake and localization of the nanocomposite which leads to the improvement of pharmacological activities. There is little knowledge about cellular uptake of nanocomposites, especially for how nanocomposites translocate across cell membranes across the cancer cells’ microenvironment. The main objective of this work is to use nanocomposites (GOP-PCA and GOP-PCA-FA) as drug delivery systems for human hepatocellular carcinoma cell line (HepG2), and to gain understanding of the cellular uptake, accumulation, and localization of the nanocomposite to enhance their activity and decrease their side-effects.

## 2. Results

### 2.1. Chrystallographic Characterisation of Nanoparticles and Cellular Morphological Changes Following Incubation with Nanoparticles Evaluated by High Resolution Transmission Electron Microscope (HR-TEM)

**Figure 1:**
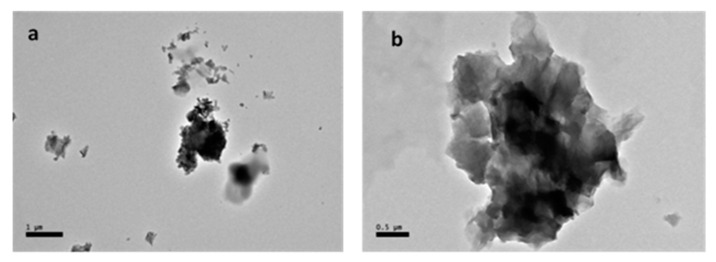
High resolution transmission electron micrographs of GOP-PCA nanocomposite under different magnification (**a**) 3000× and (**b**) 5000×. Abbreviations: GOP-PCA—graphene oxide coated PEG and loaded with protocatechuic acid. Scale bar: (**a**) 1 µm, (**b**) 0.5 µm.

**Figure 2:**
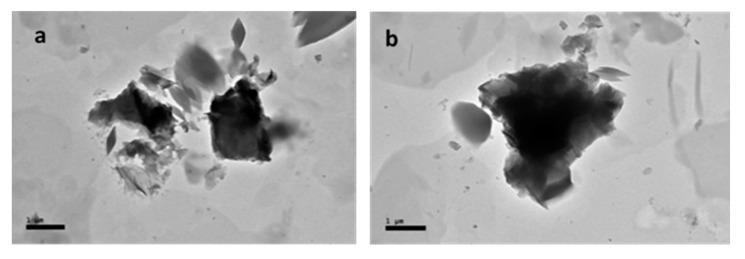
High resolution transmission electron micrographs of GOP-PCA-FA nanocomposite under different magnification (**a**) 3000× and (**b**) 5000×. Abbreviations: GOP-PCA-FA—graphene oxide coated PEG and loaded with protocatechuic acid tagged with folic acid. Scale bar: (**a**) 1 µm, (**b**) 1 µm.

The HR-TEM photomicrograph revealed shapes of GOP-PCA (Figure 1) and GOP-PCA-FA (Figure 2) shows nanocomposites under different magnification which further can be observed inside the cells.

**Figure 3:**
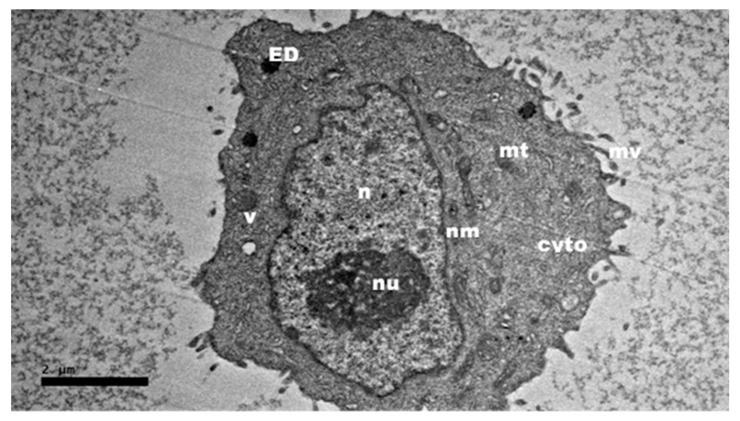
High resolution transmission electron micrographs of HepG2 cell. Untreated HepG2 cell as control for the nanocomposite cellular uptake by HR-TEM at magnification of 2000×. Abbreviations: nu—nucleolus, n—nucleus, nm—nuclear membrane, mt—mitochondria, v—vacuole, cyto—cytoplasm and ED—electron dense. Scale bar: 2 µm.

In Figure 3 the prominent intracellular features of an untreated HepG2 cell served as a control. The typical structure of a cytoplasm with various organelles including the nucleolus, nucleus and nuclear membrane areas which were also conserved well within the uniform distribution of euchromatin. Cytoplasm, appearing enriched by the presence of mitochondria, was identified, as abundant electron-dense matrixes filled their inner compartments. Less vacuole was observed, and microvilli were abundantly present in the untreated HepG2 cell.

**Figure 4:**
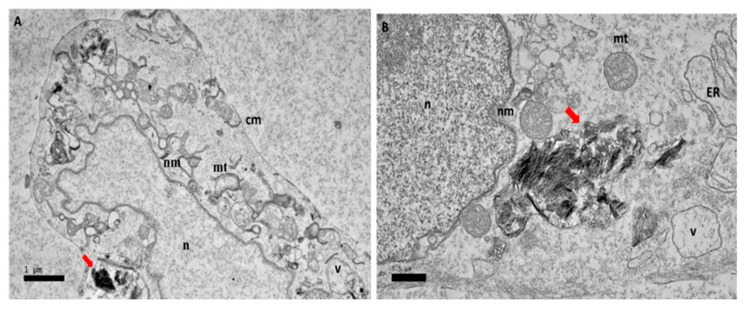
High resolution transmission electron micrographs of GOP-PCA nanocomposite at 24 h. Cellular uptake of GOP-PCA in HepG2 cell at 24 h by magnification of (**A**) 3000× and (**B**) 5000×. Abbreviations: GOP-PCA—graphene oxide coated PEG and loaded with protocatechuic acid, cm— cell membrane, n—nucleus, nm—nuclear membrane, mt—mitochondria, v—vacuole, ER— endoplasmic reticulum. Red arrow indicates the nanocomposite. Scale bar: (**A**) 1 µm (**B**) 0.5 µm.

After 24 h of exposure to GOP-PCA with HepG2, Figure 4A shows the nanocomposite entering the HepG2 cell. In Figure 4B, the nanocomposite has already entered the cytoplasm of the cells. This shows that the cellular uptake happened even before the 24 h. By observation, the mitochondria were still healthy and formation of the vacuole had happened and the double lipid bilayer of nuclear membrane was still visible.

**Figure 5:**
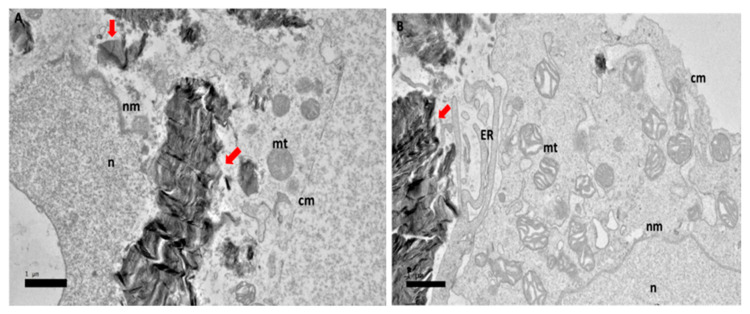
High resolution transmission electron micrographs of GOP-PCA nanocomposite at 48 h. Cellular uptake of GOP-PCA in HepG2 cell at 48 h by magnification of (**A**) 3000× and (**B**) 5000×. Abbreviations: GOP-PCA—graphene oxide coated PEG and loaded with protocatechuic acid, cm—cell membrane, n—nucleus, nm—nuclear membrane, mt—mitochondria, v—vacuole, ER—endoplasmic reticulum. Red arrow indicates the nanocomposite. Scale bar: (**A**) 1 µm (**B**) 1 µm.

**Figure 6:**
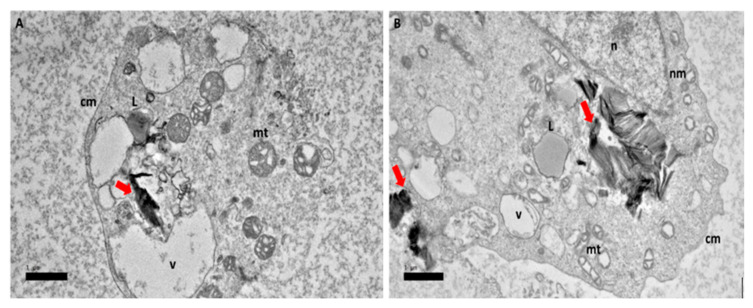
High resolution transmission electron micrographs of GOP-PCA nanocomposite at 72 h. Cellular uptake of GOP-PCA in HepG2 cell at 72 h by magnification of (**A**) 2000× and (**B**) 3000×. Abbreviations: GOP-PCA—graphene oxide coated PEG and loaded with protocatechuic acid, cm—cell membrane, n—nucleus, nm—nuclear membrane, mt—mitochondria, v—vacuole, L—lipid droplet, Red arrow indicates the nanocomposite. Scale bar: (**A**) 1 µm (**B**) 1 µm.

At 48 h (Figure 5) there has been a morphological change of the mitochondria. Irregular shapes of mitochondria were observed with the cristae loosely packed. The lipid layer of the nuclear membrane thickened. The presence of GOP-PCA, which was visible in a nanosheet shape was observed to have increased and is indicated by the red arrow (Figure 5). The final 72 h showed more prominent changes in the mitochondria where it started to lose shape and loss of cristae, and formation of numerous smaller vacuoles and formation of lipid droplets (Figure 6).

**Figure 7:**
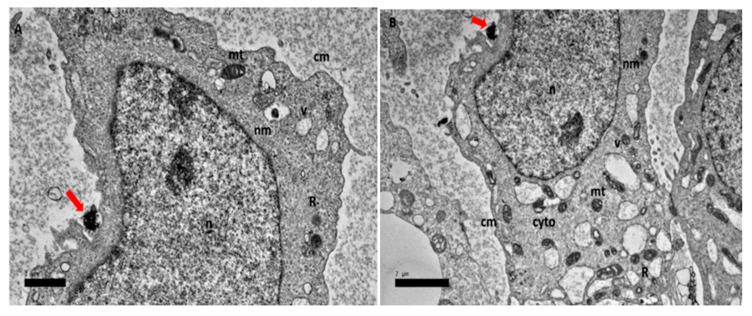
High resolution transmission electron micrographs of GOP-PCA-FA nanocomposite at 24 h. Cellular uptake of GOP-PCA-FA in HepG2 cell at 24 h by magnification of (**A**) 3000× and (**B**) 2000×. Abbreviations: GOP-PCA-FA—graphene oxide coated PEG and loaded with protocatechuic acid tagged with folic acid, cm—cell membrane, n—nucleus, nm—nuclear membrane, mt—mitochondria, v—vacuole, R—ribosomes, and cyto—cytoplasm, Red arrow indicates the nanocomposite. Scale bar: (**A**) 1 µm (**B**) 2 µm.

Figure 7 depicts GOP-PCA-FA entry into HepG2 cell via endocytosis at 24 h. The cytoplasm was compactly packed with mitochondria, numerous vacuoles, free ribosomes, nucleus and nucleolus.

**Figure 8:**
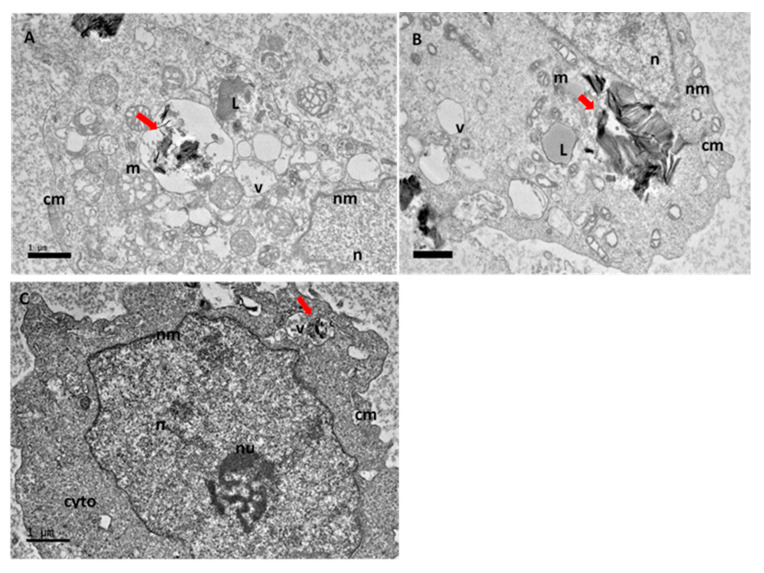
High resolution transmission electron micrographs of GOP-PCA-FA nanocomposite at 48 h. Cellular uptake of GOP-PCA-FA in HepG2 cell at 48 h by magnification of (**A**) 3000×, (**B**) 2000× and (**C**) 3000×. Abbreviations: GOP-PCA-FA—graphene oxide coated PEG and loaded with protocatechuic acid tagged with folic acid, cm—cell membrane, Nu—nucleolus, n—nucleus, nm—nuclear membrane, mt—mitochondria, v—vacuole, L—lipid droplet, and cyto—cytoplasm, Red arrow indicates the nanocomposite. Scale bar: (**A**) 1 µm (**B**) 1 µm (**C**) 1 µm.

At 48 h (Figure 8) after internalization of GOP-PCA-FA treatment, HepG2 cell demonstrated the characteristic morphological changes of apoptosis, more vacuoles started to appear, the mitochondria began to alter the shape and chromatin condensation was observed. There were also lipid droplets which were detected.

**Figure 9:**
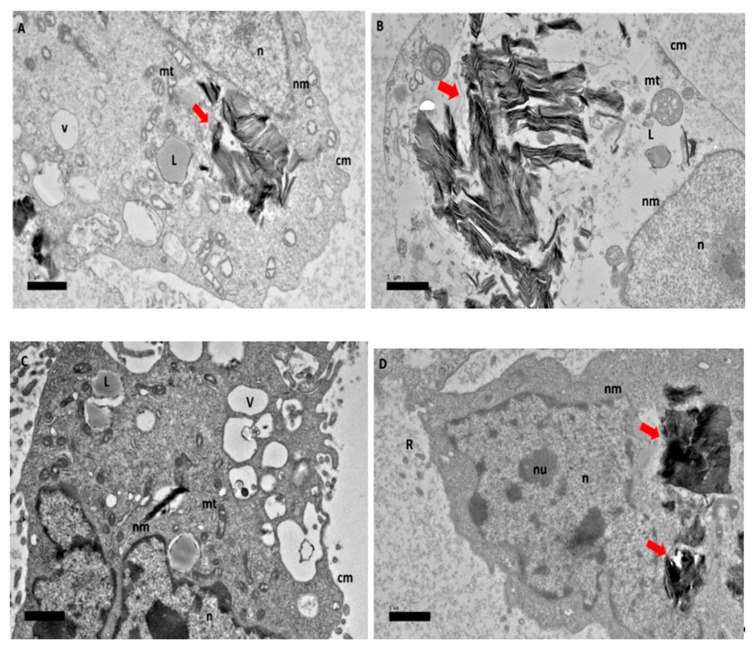
High resolution transmission electron micrographs of GOP-PCA-FA nanocomposite at 72 h. Cellular uptake of GOP-PCA-FA in HepG2 cell at 72 h by magnification of (**A**) 3000× and (**B**) 3000×, (**C**) 3000× and (**D**) 3000×. Abbreviations: GOP-PCA-FA—graphene oxide coated PEG and loaded with protocatechuic acid tagged with folic acid, cm—cell membrane, nu—nucleolus, n—nucleus, nm—nuclear membrane, mt—mitochondria, v—vacuole, L—lipid droplet, R—ribosome and cyto—cytoplasm. Red arrow indicates the nanocomposite. Scale bar: (**A**) 1 µm (**B**) 1 µm (**C**) 1 µm (**D**) 1 µm.

In the final 72 h (Figure 9A,B) changes including the formation of numerous smaller vacuoles, vacuolization of mitochondria and loss of cristae, altered cytoplasm and lipid droplets accumulation were seen. Finally, Figure 9C,D show morphological features of apoptosis, such as the formation and size of vacuole increase, presence of lipid droplets and chromatin condensation.

### 2.2. In Vitro Visualization and Localization of FITC-Labelled GOP-PCA-FA in HepG2 Cell

**Figure 10:**
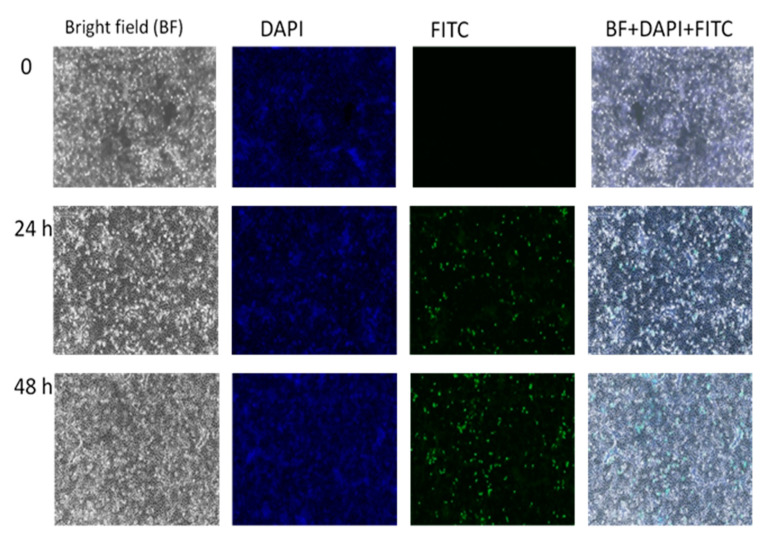
HepG2 treated with FITC-GOP-PCA-FA conjugated at 0 h, 24 h and 48 h under fluorescent microscope. Magnification 100×. Abbreviations: FITC-GOP-PCA-FA—fluorescein isothiocyanate-graphene oxide coated PEG and loaded with protocatechuic acid tagged with folic acid. Scale bar: 100 µm.

In Figure 10, the FITC-GOP-PCA-FA nanocomposite had accumulated in HepG2 and localized. The nucleus was stained with DAPI, observed as blue staining clearly depicted that cells were alive and viable. The FITC labelled nanocomposite was observed as discrete green dots. The release profile and accumulation of FITC-GOP-PCA-FA as the encapsulated cargo model was visualized at three specific time intervals: 0 h, 24 h, and 48 h using fluorescence microscopy. The observation of green fluorescence was prominent at 24 h and 48 h of post-treatment with FITC-GOP-PCA-FA nanocomposite. The green fluorescence was seen in the cytoplasm. Overlap of both signals of DAPI (blue) and FITC (green) confirmed the localization occurred in the same cells.

**Figure 11:**
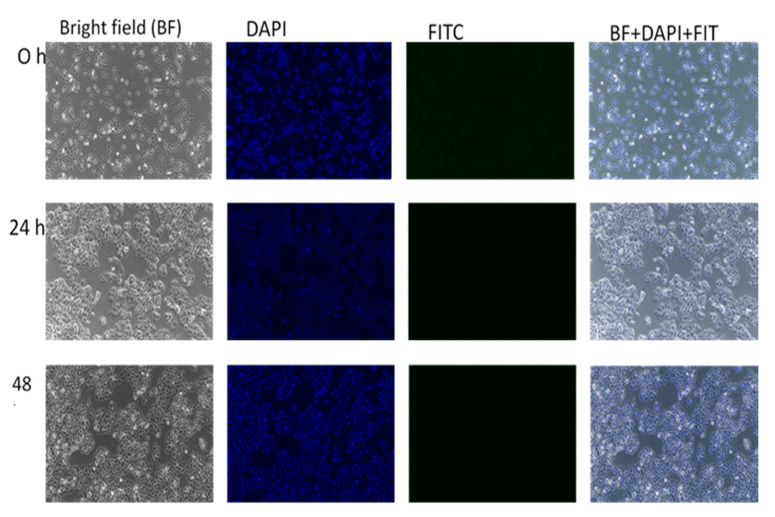
Untreated HepG2 at 0 h, 24 h and 48 h under fluorescent microscope. Magnification 100×. Scale bar: 100 µm.

**Figure 12:**
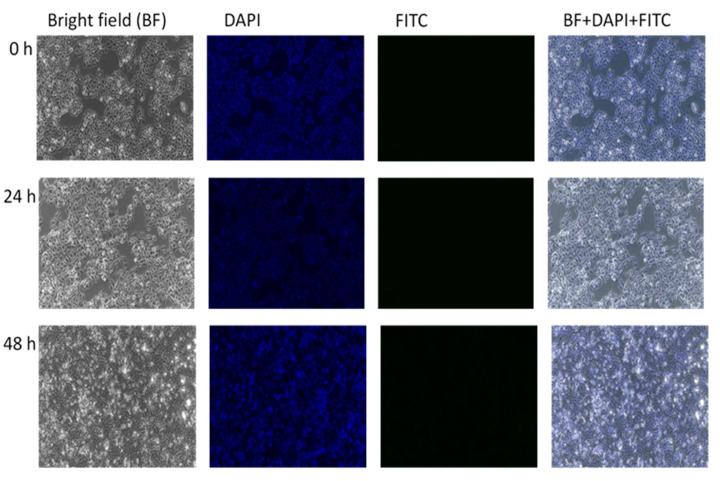
HepG2 treated with GOP-PCA-FA conjugated at 0 h, 24 h and 48 h under fluorescent microscope. Magnification 100×. Abbreviation: GOP-PCA-FA—graphene oxide coated PEG and loaded with protocatechuic acid tagged with folic acid. Scale bar: 100 µm.

**Figure 13:**
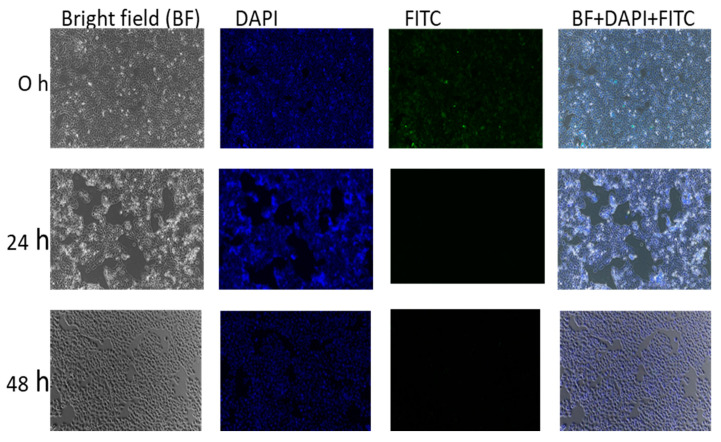
HepG2 treated with FITC alone at 0 h, 24 h and 48 h under fluorescent microscope. Magnification 100×. Abbreviation: FITC—fluorescein isothiocyanate. Scale bar: 100 µm.

Figure 11 serves as the control, where there was no treatment introduced to the HepG2 cell and the in vitro accumulations were observed at different points of time. No green fluorescence was observed in the untreated cells, indicating that no inherent autofluorescence was exhibited by the cells. In Figure 12, HepG2 cells were treated with GOP-PCA-FA nanocomposites alone. This was also an indication that there was no fluorescence exhibited with GOP-PCA-FA nanocomposite. Figure 13 shows HepG2 cells that were treated with FITC dye only. There was no visualization of fluorescence during the incubation period. The absence of a green fluorescence label in the untreated cell (Figure 11), FITC (Figure 12) and GOP-PCA-FA treated cells (Figure 13) suggested that the physical encapsulation of FITC to GOP-PCA-FA was a prerequisite for efficient accumulation and localization into cells as a potent delivery nanocarrier.

### 2.3. Influence of Nanocomposites on Cell Migration in HepG2 Cells

**Figure 14:**
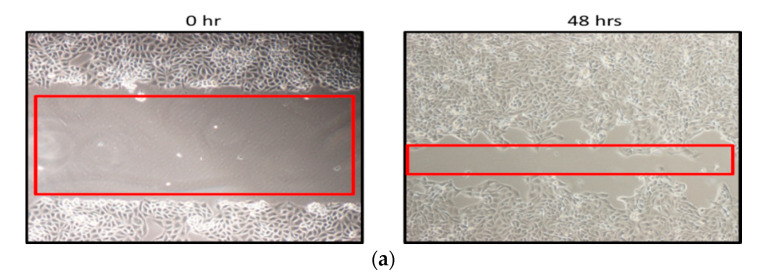

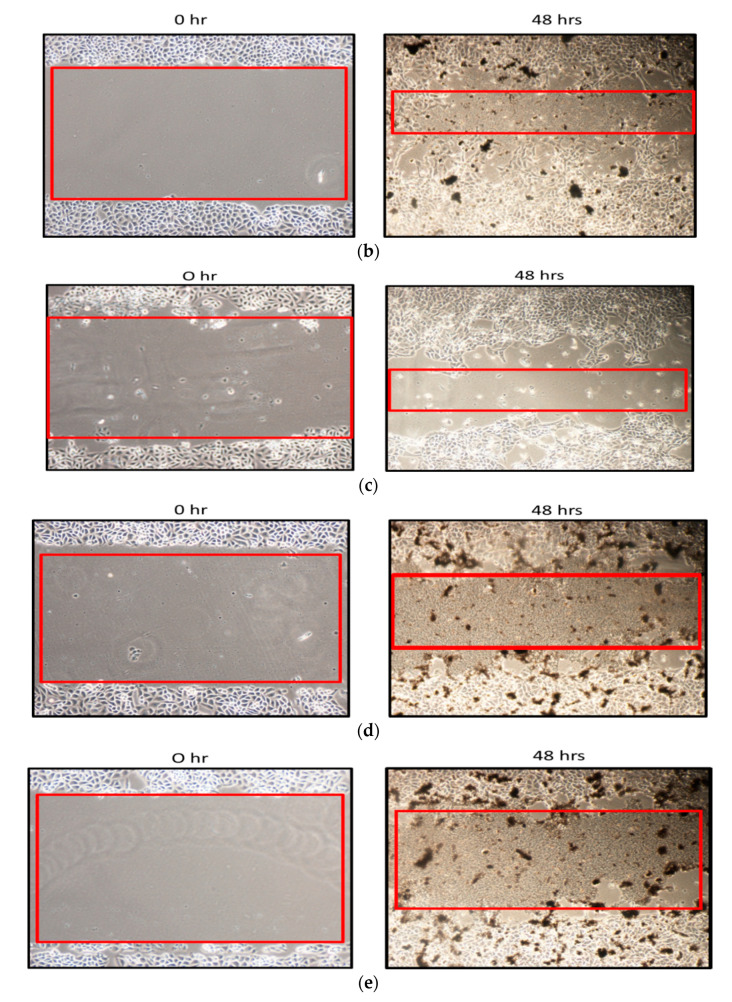
(**a**) Microscopic image evaluation of the untreated HepG2 migration assay. Magnification 40×. Scale bar: 100 µm. (**b**) Microscopic image evaluation of GOP-treated HepG2 migration assay. Magnification 40×. Scale bar: 100 µm. (**c**) Microscopic image evaluation of PCA-treated HepG2 migration assay. Magnification 40×. Scale bar: 100 µm. (**d**) Microscopic image evaluation of GOP-PCA-treated HepG2 migration assay. Magnification 40×. Scale bar: 100 µm. (**e**) Microscopic image evaluation of GOP-PCA-FA-treated HepG2 migration assay. Magnification 40×. Red box: Indication for inhibition zone. Scale bar: 100 µm. The effect of the control (untreated), GOP, PCA, GOP-PCA and GOP-PCA-FA with migration assay using a confluent monolayer of HepG2 cells at 38 µg/mL. Cell migration was observed in response to an artificial injury. A single representative area is shown immediately after 48 h treatment with the GOP, PCA, and GOP-PCA and GOP-PCA-FA nanocomposites. Abbreviation: GOP—graphene oxide coated with PEG, PCA-protocatechuic acid, GOP-PCA—graphene oxide coated PEG and loaded with protocatechuic acid, and GOP-PCA-FA—graphene oxide coated PEG and loaded with protocatechuic acid tagged with folic acid.

**Figure 15:**
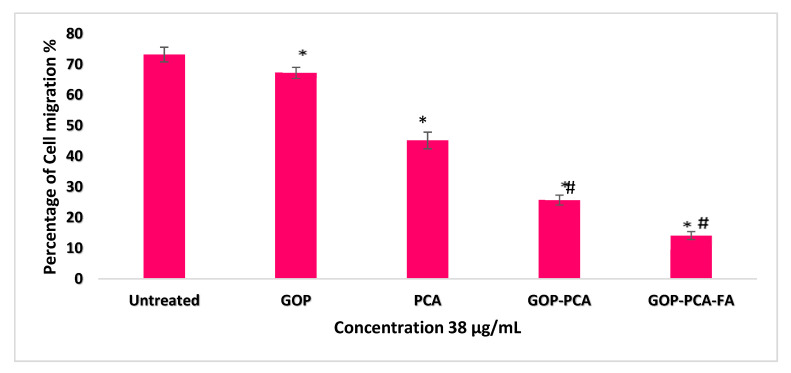
Potential cell migration rate of untreated, GOP, PCA, GOP-PCA and GOP-PCA-FA in HepG2 cells at 48 h. The triplicate data are presented as mean ± SD. The significant differences (*p* < 0.05) * were determined among untreated HepG2 against GOP, PCA, GOP-PCA and GOP-PCA-FA and (*p* < 0.05) ^#^ using PCA treated against nanocomposites by using one-way ANOVA followed by Games−Howell post hoc tests. Abbreviation: GOP—graphene oxide coated with PEG, PCA—protocatechuic acid, GOP-PCA—graphene oxide coated PEG and loaded with protocatechuic acid, GOP-PCA-FA—graphene oxide coated PEG and loaded with protocatechuic acid tagged with folic acid.

The effect of the PCA, GOP-PCA and GOP-PCA-FA nanocomposites on the migration of HepG2 cell was investigated in the cell migration assay (Figure 14a–e). Figure 14a exhibits the HepG2 cell incubated in RMPI culture media and cell migration ability from 0 h to 48 h of incubation period. This showed that there was an increase in the cell migration signal of about 73.13 ± 2.4%. Figure 14b shows the cell treated with GOP nanocarrier had significant migration of 67.24 ± 3.1% compared to the initial (0 h) untreated cell. While PCA (Figure 14c), GOP-PCA (Figure 14d) and GOP-PCA-FA (Figure 14e) showed migration of 46.13 ± 2.7%, 27.76 ± 3.4% and 16.17 ± 2.6%, respectively; which were lower percentages compared to the (0 h) untreated cell. HepG2 cells which were not treated with the nanoparticles or pristine PCA, had shown a higher percentage of cell migration while cells treated with PCA, GOP-PCA and GOP-PCA-FA induced a lesser percentage of cell migration compared to untreated HepG2. Figure 15 depicts the normalized percentage of migration in HepG2 cell line and the statistical analysis for the migration assay.

## 3. Discussion

Cellular uptake and accumulation of nanocomposite is an important aspect in drug delivery systems for an effective outcome of the drug treatment. According to TEM, the micrographs demonstrated that both GOP-PCA and GOP-PCA-FA were located intracellularly, suggesting possible uptake of nanocomposites through the plasma membrane of the HepG2 cell which resulted in altered cell morphology and an amplification of apoptotic cells. Cellular uptake of GOP-PCA was postulated due to the physiochemical characteristic where the size of the nanocomposites was around 12 nm and the PEGylation which increased the circulation time [5]. The images from TEM suggested that nanocomposites may have entered HepG2 cells via the endocytosis process [6]. The uptake of nanocomposite may have been mediated by endocytosis involving the formation of clathrin-coated endocytotic vesicles that were approximately 100 nm in diameter [7]. Folic acid conjugated nanocomposites specifically targeting the tumour cell using a receptor-mediated endocytosis mechanism which used clathrin endocytosis systems for specific transport [8]. Nanocomposite cellular uptake image analysis from HR-TEM confirmed the penetration of GO through plasma membrane and its internalization into the cytoplasm, mitochondria and nucleus. It was reported that GO penetrated cells by piercing and mechanically disrupting plasma membranes and aggregating inside the cells [9].

Cytoplasmic changes seen with TEM were accredited to the characteristics associated with apoptosis. In addition, the nanocomposite-treated HepG2 cell displayed condensed cristae of mitochondria as a typical morphological attribute to apoptosis which was observed after 48 h. In the course of apoptosis, mitochondria undergo excessive structural changes that lead to increases in the release of cytochrome c and other apoptotic proteins from the intermembrane space or intercristal space. According to Sesso et al., 2012, reported the rapid loss of selective permeability of the inner mitochondrial membrane would cause cytoplasmic fluids to traverse, thus resulting in swelling of the mitochondria matrix [10]. The outer mitochondrial membrane which has a small surface ratio compared to the inner mitochondrial membrane, would cause cell rupture, releasing cytochrome c into the cytoplasmic region [11].

The localization and accumulation of GOP-PCA-FA conjugated FITC served as confirmation for real-time imaging to confirm the nanodrug was delivered at the targeted site. FITC (fluorescein isothiocyanate) is a rapid, simple, and sensitive fluorescein used to quantify cell-associated study by fluorometer. FITC is a widely used fluorochrome, conjugated due to its improved aqueous solubility and photostability [12]. From Figure 10, FITC-GOP-PCA-FA confirmed that localization of the GOP-PCA-FA occurred inside the HepG2 cells after 48h of incubation. Similar observations were made when functionalized graphene oxide coated transferrin conjugated with FITC were incubated for 48 h in glioma U251 cells observed under fluorescent imaging [13]. This observation has suggested that GOP has the ability to deliver the drug PCA into HepG2 cells once they intracellularly accumulated.

The migration ability of tumor cells is renowned to be a key step in tumor progression [14]. Inhibition of cell migration is one of the factors in successful anticancer treatment and ensuring a relatively longer survival period for patients. Cell migration assay in Figure 14 shows that GOP-PCA-FA nanocomposite had significant inhibition of cell migration compared to pristine PCA or untreated HepG2 cells. The inhibition of cell migration may occur through impairment of oxidative phosphorylation and mitochondrial respiration [15]. This observation was supported by a study where treatment with nanographene oxide-PEG-dendrimer/anti-miR-21 strongly inhibited lung cancer cell migration and invasion [16]. Therefore, the potential of GOP-PCA-FA for the inhibition of effective migration in HepG2 cancer cells deserves further in vivo evaluation.

## 4. Material and Methods

### 4.1. Materials

The cell that was chosen for this experiment was HepG2 (ATCC HB 8065) (Homo sapiens) cell line obtained from liver epithelial cells of a 15-year-old adolescent Caucasian male with a hepatocellular carcinoma (HCC). The 4,6-diamidino-2-phenylindole dihydrochloride (DAPI), and fluorescein 5(6)-isothiocyanate (FITC) were purchased from Sigma Aldrich (St Louis, MO, USA) and utilized without further purification. Diethyl ether, sodium hydroxide, hydrochloric acid (HCl, 37%), ethyl alcohol (99.7% *v/v*), 37% formaldehyde, and acetic acid glacial were bought from Friedemann Schmidt (Parkwood, WA, USA). Protocatechuic acid (AK Scientific, Union City, CA, USA). The nanocomposites were obtained from a previous study GOP (Graphene oxide+PEG), GOP-PCA (Graphene oxide+PEG+protocatechuic acid), GOP-PCA-FA (Graphene oxide+PEG+protocatechuicacid+folic acid) [5].

### 4.2. Preparation of Cell Lines

All cancer cells lines were prepared using guidance from the manufacturer’s instructions using Roswell Park Memorial Institute (RPMI) medium supplemented with 10% fetal bovine serum (FBS), penicillin and streptomycin 1%. The cells were incubated and grown at 37 °C in a humidified 5% CO^2^ incubator. After 18−20 h, once the cells reached confluency by forming a monolayer on the cell culture flask, the supernatant was discarded and the cells were incubated with trypsin-EDTA (ethylenediaminetetraacetic acid) for 5−10 min (depending on the cells) to detach the cells. The determination of cell viability was determined using trypan blue exclusion assay and cell count using a haemocytometer.

### 4.3. Trypan Blue Exclusion Assay

Once the cell pellets were resuspended in cell culture medium, a 50 µL sample of the cell suspension was put in a 1.5 mL centrifuge tube. An equal amount of trypan blue dye was added and mixed thoroughly. A total of 10 µL of the mixture was removed by pipette and placed on the haemacytometer counter and covered with a cover slip to avoid air bubbles. The haemocytometer was viewed under a light microscope and the number of viable cells (clear cells) from each large square was counted. The final value of the viable cells/ mL in the cell suspension calculated according to the Equation (1):

Number of cells counted × dilution factor Total volume of the cells counted(1)

### 4.4. Morphological Observation through High Resolution Transmission Electron Microscope (HR-TEM)

The HepG2 cells were cultured overnight in a 25 cm^2^ culture flask with approximately 1.0 × 10^5^ cells/mL and then treated with IC_50_ of GOP-PCA (29.98 μg/mL) and GOP-PCA-FA (18.89 μg/mL) [5] nanocomposites for an incubation period 24, 48 and 72 h. Some HepG2 cells incubated with RPMI media only were used as the negative control. The cell pellets were centrifuged and fixed with 2.5% glutaraldehyde in 0.1 M sodium cacodylate buffer for 2 h at 4 °C. Following fixation, the cell pellets were washed with 0.1 M sodium cacodylate buffer and postfixed in 1% aqueous osmium tetroxide for 2 h at 4 °C. The samples were then diced into 1 mm cubes and dehydrated in a series of acetone (v:v 30%, 50%, 75%, 80%, 90%, 95% and 100%). The samples were initially infiltrated with a 1:1 mixture of acetone and resin for 2 h and subsequently infiltrated with 100% resin overnight. The samples were transferred into BEEM capsules and polymerized at 60 °C for 48 h. Ultrathin sections (1.0 A) of the resin-embedded tissue samples were obtained by using the ultramicrotome and transferred onto 400 mesh copper grids. Samples were subsequently stained with uranyl acetate and lead citrate. The stained sections were finally examined under the HR-TEM (JEOL JEM-2100F, Tokyo, Japan) transmission electron microscope.

### 4.5. Synthesis of FITC-Labelled GOP-PCA-FA

FITC-labelled GOP-PCA-FA nanocomposites were prepared by dissolving 1 mg FITC powder into 1 mL DMSO as a stock solution. Approximately 50 μL of the FITC solution was then added to 200 μL of GOP-PCA-FA nanocomposite and incubated for 20 min at 24 °C. The process had to be conducted away from light since FITC is light sensitive. The total mixture was thoroughly mixed to ensure a homogeneous suspension was obtained. The FITC-GOP-PCA-FA was covered with aluminum foil to protect from exposure to light and stored at 4 °C for further analyses.

### 4.6. In vitro Cellular Uptake and Localization by Fluorescent Microscopy Analysis

The cells were seeded into six-well plates at a density of 5.0 × 10^5^ cells/well. Once confluent, cells were treated with 200 μL GOP-PCA-FA and FITC-GOP-PCA-FA and incubated for different time periods (0, 24, 48 h). The cells were washed with 1× PBS, followed by fixation using 4% formaldehyde and incubated for 3–5 min in an incubator. Then, the fixatives were discarded and washed with 1× PBS. The cell nucleus was stained with DAPI solution (0.5 μg/mL). The cells were then further incubated for 5 min at room temperature. Finally, the DAPI stain was discarded and washed with 1× PBS. Fresh culture medium was added. The localization and accumulation of FITC-GOP-PCA-FA was observed under an IX3P2F/Olympus fluorescence inverted microscope (Olympus, Hamburg, Germany).

### 4.7. Cell Migration Assay

The migration assay was conducted to assess the cell exclusion zone. The HepG2 cells were seeded in six-well plates with a density of 1 × 10^5^ cells/well. After the cells reached confluence of 90%, a scratch was made on the cell monolayer with 20 µL pipette tips. Fresh cell culture medium containing PCA, GOP-PCA and GOP-PCA-FA at a concentration of 38 μg/mL (IC_50_ of PCA) was introduced to the cells. Cell migration was analyzed after 48 h with an inverted microscope (Olympus Soft Imaging Solutions). The image was captured immediately after scratching and after 48 h of treatment. The area of migration was calculated using Image J (1.51k software). All experiments were repeated at least three times.

## 5. Conclusions

In conclusion, visualization from TEM micrographs demonstrated and identified the morphological changes, cellular uptake and localization of GOP-PCA and GOP-PCA-FA nanocomposites in the HepG2 cell. From observation, it can be postulated that the nanocomposite uptake mechanism, localization and their potential therapeutic impact or toxicity is unique because of the unique features of individual nanocomposites, variations in cell membrane properties, and difference between tissues depending on disease conditions. It is also important to correlate and for data obtained in vitro to be translated in vivo. Better understanding and background knowledge on the nanocomposite uptake mechanism and localization is needed for the development and design of an effective nanocomposite for biomedical applications. Moreover, real-time imaging of live cells helps in understanding the basic biological phenomenon of the organism. Fluorescent probe nanocomposites used in vitro and in vivo as contrast agents aiding in pharmaceutical, therapeutic and diagnostic areas in the medical field shown in the visualization and internalization of the nanocomposites. The fluorescent particles are proposed as highly efficient nanomaterial tags and can be directed for diagnostic targeting in surface functionalization. This study allows us to visualize more accurate therapeutic delivery and diagnostic imaging based on fluorescent tagging of nanocomposites in the near future.

## Abbreviations

GOP	Graphene oxide+PEG
GOP-PCA	Graphene oxide+PEG+protocatechuic acid
GOP-PCA-FA	Graphene oxide+PEG+protocatechuicacid+folic acid
DAPI	4,6-diamidino-2-phenylindole dihydrochloride
FITC	fluorescein 5(6)-isothiocyanate
